# Scientific Items

**Published:** 1887-07

**Authors:** 


### SCIENTIFIC ITEMS.

**The Living Earth.**—As another illustration of the life that dwells in nature, let us briefly consider earthquakes. The peculiar terror of an earthquake lies mainly in the suddenness of its approach. Volcanic eruptions are usually preceded by vast rumblings, or jets of steam, or other unmistakable tokens. Hurricanes and cyclones, in like manner, have heralds that announce their coming. But with an earthquake there are no premonitory symptoms. The great earthquake which took place at Lisbon in the year 1755 found the people engaged in their ordinary occupations. All the shocks were over in about five minutes. The first shock lasted about six seconds. In that brief space of time most of the houses had been thrown down and thousands of men, women and children crushed beneath the ruins. At times the ocean lends fresh terrors to the scene. Thus at Lisbon a wave of water over fifty feet high rushed in among the houses, and covered what still remained. In the island of Jamaica, on a different occasion, two thousand five hundred houses were buried in three minutes under thirty feet of water. Recent delicate scientific experiments have discovered the fact that the surface of the land is never absolutely at rest for more than thirty hours at a time. Thus those great earthquakes which make epochs in history are merely extreme cases of forces that seldom sleep.—Extract from a lecture by A. Ewbank in *Indian Engineer*, published in Calcutta.—*Scientific American.*

**A Field of Work under the Sea.**—A writer in one of our contemporaries suggests the development of submarine navigation as one of the works of the future. He contrasts the amount of time and thought which has been expended upon the solution of the problem of flight with the little that has been done in the other field. Men have ever shown themselves more anxious to rival the birds than to cope with the element of the fishes. Dædalus' flight from Crete and the fatal melting of the wings of Icarus, his fall and death, are features of one of the most famous legends of antiquity. But we do not read of Dædalus, or of any other inventor of his day, constructing a submarine vessel. Yet under the waters all is peace, where on the surface the floating ship is exposed to the maximum wave action of the unstable element. The character of instability disappears from the ocean at a small depth, and thirty or forty feet down it is the type of constancy of conditions. The prediction is formally made by the writer in question that in the future this mode of journeying will be extensively indulged in. Then Jules Verne's work will read like a prophecy, and twenty thousand leagues, and many times that, will annually be sailed under the sea. Such are substantially the conclusions of our writer. Whether they will be verified or not must be left, we fear, to future generations to see.—*Scientific American.*

**Simple Test for Wall Paper.**—A simple and easily applied test for wall papers has been devised by Mr. F. F. Grenstted. No apparatus is needed beyond an ordinary gas jet, which is turned down to quite a pin-point, until the flame is wholly blue. When this has been done, a strip of the paper suspected to contain arsenic is cut one-sixteenth of an inch wide and an inch or two long. Directly the edge of this paper is brought into contact with the outer edge of the gas flame a gray coloration, due to arsenic, will be seen in the flame (test No. 1). The paper is burned a little, and the fumes that are given off will be found to have a strong garlick-like odor, due to the vapor of arsenic acid (test No. 2.)

Take the paper away from the flames and look at the charred end—the carbon will be colored a bronze red; this is a copper reduced by the carbon (test No. 3). Being now away from the flame in a fine state of division, the copper is slightly oxidized by the air, and on placing the charred end, a second time, not too far into the flame, the flame will now be colored green by copper (test No. 4.) By this simple means it is possible to form an opinion, without an apparatus and without leaving the room, as to whether any wall paper contains arsenic, for copper arseniate is commonly used in preparing wall papers. Tests one and two would be yielded by any paper containing arsenic in considerable quantities.—*British Medical Journal.*

**Detection of Micro-Organisms.**—Since Koch devised his now well-known method of cultivating micro-organisms on plates coated with gelatine, great advances have been made in bacteriological research. Especially is this true of that branch which deals with bacteria in drinking-water. Dr. Frankland has found that, in the storage and filtration to which London water is subjected, the number of micro-organisms is reduced ninety-five per cent. Dr. Bolton has shown that the spores of anthrax remain alive in distilled water for ninety days, and in polluted well water for a year, while the bacilli themselves are very short lived. The coma bacillus of Koch, as is known, will reproduce itself in water. The importance of these observations is evident when it is considered that, regarding the germ theory as true, zymotic diseases may be spread by means of water impregnated with their germs.—*Science.*—*Microscope.*

**It** is confidently predicted that in five years the magnesium light will be as familiar as is the electric light to-day. Its high cost has heretofore been a serious obstacle, but that is said to be now removed by a new German process which has reduced the price from $40 to $8 a pound, with a prospect of still further cheapening. A wire of moderate size equals the light of seventy-five steraine candles; the cost is now but little more than gas.—*Ex.*

**The** bark of the Takenaya, a New Zealand coniferous tree, is used for dyeing yellow in Otago, and is now being introduced in London to give a deep golden yellow to "dogskin" gloves.

